# Single-Cell RNA-Seq Analysis Links DNMT3B and PFKFB4 Transcriptional Profiles with Metastatic Traits in Hepatoblastoma

**DOI:** 10.3390/biom14111394

**Published:** 2024-10-31

**Authors:** Christophe Desterke, Raquel Francés, Claudia Monge, Agnès Marchio, Pascal Pineau, Jorge Mata-Garrido

**Affiliations:** 1 Faculté de Médecine du Kremlin Bicêtre, Université Paris-Sud, Université Paris-Saclay, 94270 Le Kremlin-Bicêtre, France; christophe.desterke@inserm.fr; 2 Cell and Tissue Biology Group, Anatomy and Cell Biology Department, University of Cantabria-IDIVAL, 39011 Santander, Spain; 3 International Joint Laboratory of Molecular Anthropological Oncology (LOAM), IRD, INEN, Lima 15038, Peru

**Keywords:** hepatoblastoma, metastasis, CHIC risk, metabolism, epigenetics, DNA methylation, glycolysis, transcriptome

## Abstract

Hepatoblastoma is the most common primary liver cancer in children. Poor outcomes are primarily associated with patients who have distant metastases. Using the Mammalian Metabolic Enzyme Database, we investigated the overexpression of metabolic enzymes in hepatoblastoma tumors compared to noncancerous liver tissue in the GSE131329 transcriptome dataset. For the overexpressed enzymes, we applied ElasticNet machine learning to assess their predictive value for metastasis. A metabolic expression score was then computed from the significant enzymes and integrated into a clinical-biological logistic regression model. Forty-one overexpressed enzymes distinguished hepatoblastoma tumors from noncancerous liver tissues. Eighteen of these enzymes predicted metastasis status with an AUC of 0.90, demonstrating 85.7% sensitivity and 92.3% specificity. ElasticNet machine learning identified *DNMT3B* and *PFKFB4* as key predictors of metastasis. Univariate analyses confirmed the significance of these enzymes, with respective *p*-values of 0.0058 and 0.0091. A metabolic score based on *DNMT3B* and *PFKFB4* expression discriminated metastasis status and high-risk CHIC scores (*p*-value = 0.005). The metabolic score was more sensitive than the C1/C2 classifier in predicting metastasis (accuracy: 0.72 vs. 0.55). In a regression model integrating the metabolic score with epidemiological parameters (gender, age at diagnosis, histological type, and clinical PRETEXT stage), the metabolic score was confirmed as an independent adverse predictor of metastasis (*p*-value = 0.003, odds ratio: 2.12). This study identified the dual overexpression of *PFKFB4* and *DNMT3B* in hepatoblastoma patients at risk of metastasis (high-risk CHIC classification). The combined tumor expression of *DNMT3B* and *PFKFB4* was used to compute a metabolic score, which was validated as an independent predictor of metastatic status in hepatoblastoma.

## 1. Introduction

Hepatoblastoma is the most common primary liver cancer in children. Before the 1980s, complete tumor resection was the only curative treatment for children with primary liver tumors. In recent decades, the introduction of chemotherapy has improved patient management, but outcomes remain poor for those with distant metastases [1,2].

The clinical evaluation of hepatoblastoma includes several well-established prognostic factors, such as the presence of metastatic disease at diagnosis, the pre-treatment extent of the tumor (PRETEXT stage), histological subtypes, and serum alpha-fetoprotein (AFP) levels [3,4,5]. Prognostic staging systems, such as PRETEXT, were developed to reduce the toxic side effects of chemotherapy by guiding patient treatment. The PRETEXT score is based on the number of liver segments involved, the extent of local invasion, regional lymph node involvement, and the presence of distant metastases [1,6,7]. Additionally, the Children’s Hepatic Tumor International Collaborative (CHIC) developed the CHIC risk stratification system [3,8]. The CHIC classification defines standard-risk disease as PRETEXT I/II tumors or PRETEXT III tumors without high-risk PRETEXT annotation factors. Intermediate-risk disease includes PRETEXT IV tumors or PRETEXT I-III tumors with positive PRETEXT annotation factors. High-risk disease is defined as metastatic hepatoblastoma [9].

The Warburg effect plays a crucial role in hepatoblastoma tumor development. These tumors are often associated with somatic mutations in the CTNNB1 gene, which encodes β-catenin, a transcriptional co-factor involved in Wnt signaling. In mouse models, the tumor’s metabolic profile is heavily influenced by the specific types of CTNNB1 mutations present [10]. This metabolic variation has also been observed in human hepatoblastoma cell lines, where β-catenin has been shown to regulate the GLUT3-SLC2A3 glucose transporter [11]. Moreover, brain-expressed X-linked protein 1 (BEX1) helps maintain the stem cell-like properties of hepatoblastoma cells by promoting the Warburg effect through a PPARγ/PDK1-dependent pathway [12].

Alongside these key processes, cellular metabolites provide the necessary chemical groups for DNA and histone modifications, resulting in a complex interaction between metabolism and epigenetics [10]. DNA methylation regulates many biological processes, including gene transcription, X chromosome inactivation, and genomic imprinting [11]. During tumorigenesis, disruptions in DNA methylation can repress tumor suppressor genes through hypermethylation while causing genomic instability via hypomethylation of repetitive sequences [12]. Studies of hepatoblastoma have revealed genome-wide methylation dysfunction, characterized by hypermethylation at specific CpG islands and mild hypomethylation in non-repetitive intergenic regions [13].

In this study, we identified dual overexpression of *PFKFB4* and *DNMT3B* in hepatoblastoma patient samples with metastatic risk (high-risk CHIC stratification), based on the metabolic enzyme expression program of hepatoblastoma tumors. A meta.score, computed from the combined tumor expression of *DNMT3B* and *PFKFB4*, was confirmed as an independent adverse predictor of metastatic status in hepatoblastoma. We propose that the meta.score could be useful for improving surveillance in patients at high risk of tumor recurrence (e.g., age > 3 years, PRETEXT IV).

## 2. Materials and Methods

### 2.1. Public Hepatoblastoma Tumor Transcriptome Dataset

The GSE131329 [9] transcriptome dataset was downloaded from the Gene Expression Omnibus (GEO) using the GEOquery R package (version 2.70.0) [14,15] within the R software environment (version 4.3.3). This microarray dataset can be accessed using the following link: https://www.ncbi.nlm.nih.gov/geo/query/acc.cgi?acc=GSE131329 (accessed on 3 September 2024). The experiments were performed using the Affymetrix Human Gene 1.0 ST Array ([HuGene-1_0-st]), corresponding to the annotation platform GPL6244, which is available at https://www.ncbi.nlm.nih.gov/geo/query/acc.cgi?acc=GPL6244 (accessed on 3 September 2024). The dataset includes transcriptome data from 53 hepatoblastoma tissues and 14 noncancerous liver tissue samples (see Table 1, column “Total” for a description of the tumor samples).

### 2.2. Expression of Metabolic Program

In our previous work [16], using data from the Mammalian Metabolic Enzyme Database [17], we identified a metabolic program consisting of 41 enzymes overexpressed in hepatoblastoma tumors (referred to as HB metabolic-41) (Table S1). Using the HB metabolic-41 expression program, we performed an unsupervised principal component analysis (PCA) on the total transcriptome data of 53 hepatoblastoma tissues and 14 noncancerous liver tissue samples from the GSE131329 dataset, as well as on the 53 tumor samples alone, using the FactoMineR R package (version 2.11) [18]. The expression levels of enzymes predictive of metastasis status were used for unsupervised clustering (Euclidean distances and Ward.D2 method) with the pheatmap R package (version 1.0.12). Receiver operating characteristic (ROC) curves and the area under the curve (AUC) for predicting metastasis status based on expression data were calculated using the pROC R package (version 1.18.5) [19].

### 2.3. Machine Learning ElasticNet Model of Metabolic Markers

For the tumor samples, the expression of the HB metabolic-41 program was extracted from the GSE131329 dataset and combined with the metastasis status metadata as the outcome variable. After splitting the data into training and validation sets (0.7/0.3 ratio), the ElasticNet model (binary tumor cell status outcome) was tuned for alpha and lambda parameters using the caret R package (version 6.0-94) [20]. The final ElasticNet model was fitted using the best alpha parameter (alpha = 0.2) with the glmnet R package (version 4.1-8) [21].

A loop of univariate binomial regression was then performed on each enzyme in the HB metabolic-41 program based on the metastatic binomial outcome. These analyses were conducted using the logitloop R package (version 1.0.0), available at https://github.com/cdesterke/logitloop (accessed on 3 September 2024). A meta.score was computed using the most significant metabolic genes, summing their expression and binomial beta scores according to Equation (1):

meta.score = ((DNMT3B * 3.38905474467193) + (PFKFB4 * 2.3096192085631)) (1)

Comparisons between qualitative parameters were conducted using the chi-square test in R, and the corresponding mosaic plots were generated with the vcd R package (version 1.4-12) [22]. The optimal threshold for the meta.score was determined using the cutpointr R package (version 1.1.2). A binomial regression model, with metastasis status (negative or positive) as the outcome, was constructed using the generalized linear model (“glm”) function in R with a binomial logit family. This model incorporated the metabolic expression score (a combination of *DNMT3B* and *PFKFB4* expression in tumors, referred to as the meta.score) along with several epidemiological parameters, including age at diagnosis and gender, as well as clinical (PRETEXT stage) and histological parameters (tumor histological type and state of differentiation). The corresponding nomogram for the binomial regression model was created using the regplot R package (version 1.1).

### 2.4. C1/C2 Classifier

Following the methodology from a previous publication [23], the hepatoblastoma C1/C2 16-gene classifier was applied to the GSE131329 transcriptome cohort. This classifier includes the expression of 16 genes: *GHR*, *APCS*, *C1S*, *AQP9*, *CYP2E1*, *APOC4*, *HPD*, *NLE*, *RPL10A*, *E2F5*, *BUB1*, *DLG7*, *IGSF1*, *AFP*, *DUSP9*, and *ALDH2*. K-means clustering was performed on the selected gene expression matrix to classify the cohort into two groups. The results were then evaluated using a confusion matrix and the accuracy of metastasis prediction was assessed using the caret R package (version 6.0-94) [20].

## 3. Results

### 3.1. Metabolic Program of Hepatoblastoma Tumors Predicts Distant Metastasis Status

**Figure 1:**
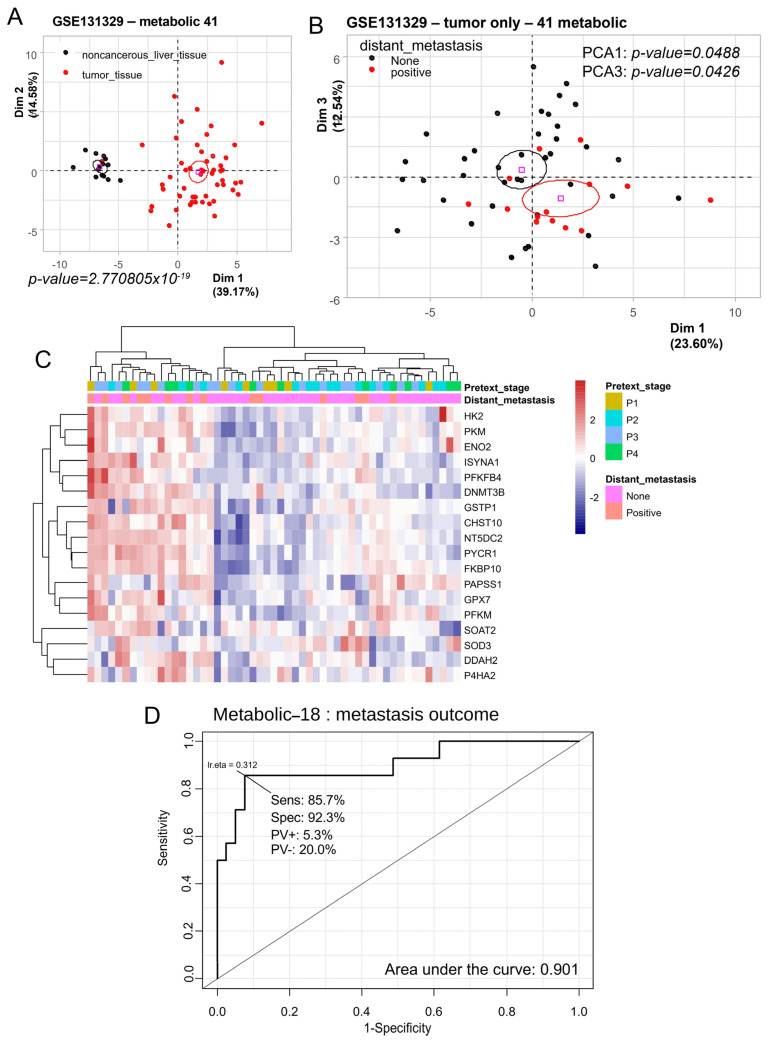
The metabolic profile of hepatoblastoma tumors predicts distant metastasis status: dataset GSE131329. (**A**) Principal component analysis (PCA) was performed on the expression of the hepatoblastoma (HB) metabolic-41 program, which was subjected to tissue type stratification (noncancerous liver tissue vs. tumor), yielding a *p*-value on the first principal axis. (**B**) PCA was also performed on the expression of the HB metabolic-41 program, stratified by metastasis status, resulting in *p*-values on the first and third principal axes. (**C**) Unsupervised clustering (using Euclidean distances) and an expression heatmap of the 18 most informative metabolic markers in HB tumors (selected based on PCA axes for metastasis prediction) are presented. (**D**) The receiver operating characteristic (ROC) curve and the area under the curve (AUC) are also shown for the expression of these 18 markers used to predict metastasis status in hepatoblastoma tumors (Sens: sensitivity, Spe: specificity, PV+: positive predictive value, PV-: negative predictive value).

Previous studies utilizing data from the Mammalian Metabolic Enzyme Database [20] identified a metabolic program comprising 41 enzymes that are overexpressed in hepatoblastoma tumor cells. In an independent transcriptome cohort (dataset GSE131329) [9] (Table 1), unsupervised principal component analysis confirmed that the HB metabolic-41 program effectively stratified noncancerous liver tissue samples from hepatoblastoma tumor samples (*p*-value = 2.77 × 10^−19^, Figure 1A).

Unsupervised principal component analysis (PCA) performed solely on the 53 tumor samples with a metabolic-41 expression program enabled the stratification of metastatic outcomes in patients (PCA1 *p*-value = 0.048 and PCA3 *p*-value = 0.042, Figure 1B). Analysis of the loadings for the respective PCA1 and PCA3 axes allowed for the identification of 18 enzymes that were overexpressed in tumors with a positive metastasis status. Unsupervised clustering (using Euclidean distances) of the expression of these 18 enzymes enabled the stratification of the majority of tumor samples according to their metastatic status (*p* = 0.0492, OR = 3.9, 95% CI = 1.1–12.1) (Figure 1C). The predictive enzymes comprised the following: *HK2* (hexokinase 2), *PKM* (pyruvate kinase M1/2), *ENO2* (enolase 2), *ISYNA1* (inositol-3-phosphate synthase 1), *PFKFB4* (6-phosphofructo-2-kinase/fructose-2,6-biphosphatase 4), *DNMT3B* (DNA methyltransferase 3 beta), GSTP1 (glutathione S-transferase pi 1), *CHST10* (carbohydrate sulfotransferase 10), NT5DC2 (5′-nucleotidase domain containing 2), *PYCR1* (pyrroline-5-carboxylate reductase 1), *FKBP10* (FKBP prolyl isomerase 10), *PAPSS1* (3′-phosphoadenosine 5′-phosphosulfate synthase 1), GPX7 (glutathione peroxidase 7), *PFKM* (phosphofructokinase, muscle), *SOAT2* (sterol O-acyltransferase 2), *SOD3* (superoxide dismutase 3), *DDAH2* (dimethylarginine dimethylaminohydrolase 2), and *P4HA2* (prolyl 4-hydroxylase subunit alpha 2). Receiver operating characteristic (ROC) curve analysis of the combined expression of these 18 enzymes allowed for the prediction of a positive metastasis status with an area under the curve (AUC) of 0.901, a sensitivity of 85.7%, and a specificity of 92.3% (Figure 1D). These results indicate that the metabolic transcriptional program is altered in hepatoblastoma tumors in the context of metastasis.

### 3.2. Computational Analysis of Metabolic Enzymes for Predicting Metastatic Potential in Hepatoblastoma

**Figure 2:**
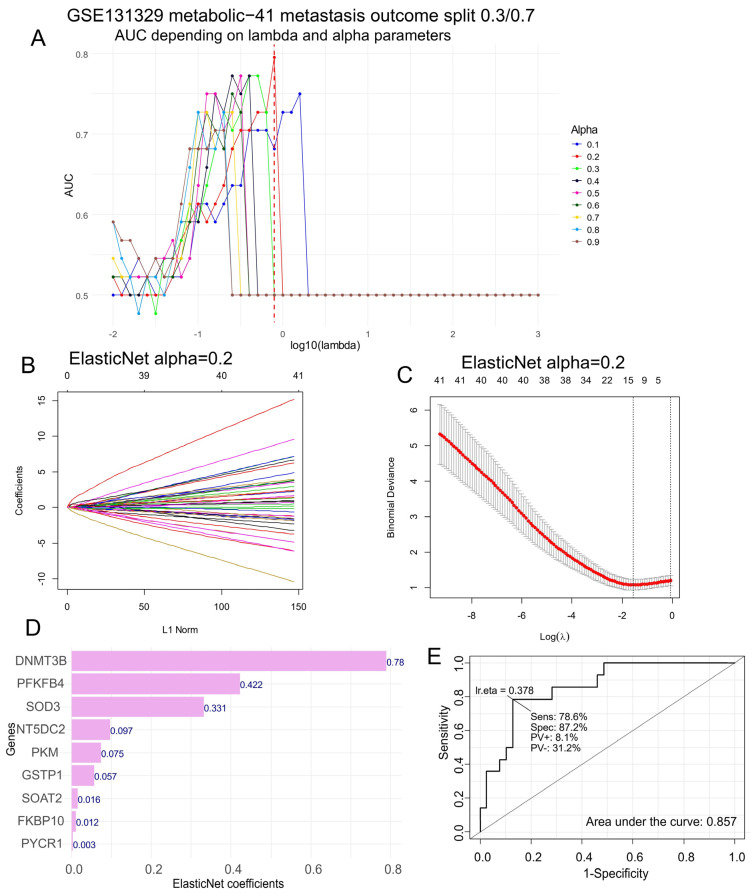
DNMT3B expression as a prognostic marker for metastasis in hepatoblastoma. We conducted ElasticNet tuning for the lambda and alpha parameters in the HB metabolic-41 expression program to predict metastasis status in tumors, utilizing an area under the curve (AUC) of 0.7/0.3 after splitting the data into training and validation cohorts. We obtained the following results: (**A**) the optimal ElasticNet tuning; (**B**) the ElasticNet fit with the best alpha parameter, fixed at 0.2; (**C**) the coefficient of variation of the ElasticNet with the best alpha parameter, fixed at 0.2; (**D**) a bar plot of the most predictive positive ElasticNet coefficients for metastasis, related to metabolic markers; and (**E**) the receiver operating characteristic (ROC) curve and area under the curve for predicting metastasis based on the combination of nine optimal ElasticNet metabolic markers: *DNMT3B*, *PFKFB4*, *SOD3*, *NT5DC2*, *PKM*, *GSTP1*, *SOAT2*, *FKBP10*, and *PYCR1*.

To evaluate the significance of each enzyme’s transcriptional expression and its correlation with metastatic status in hepatoblastoma, we employed machine learning tuning using ElasticNet on the expression data from the HB metabolic-41 expression program. We split the cohort (Table 1) into two datasets: a training dataset consisting of 70% of the samples, and a validation dataset comprising 30% of the samples. We then trained a sequence of ElasticNet models on the training set, with varied lambda and alpha parameters (Figure 2A). The best prediction in the validation set was achieved with an alpha parameter fixed at 0.2, yielding an area under the curve of 0.78 for the optimal lambda (Figure 2A). We fitted the ElasticNet model with an optimal alpha parameter fixed at 0.2 (Figure 2B) and analyzed the coefficient of variation of this model (Figure 2C). This analysis allowed us to identify nine metabolic enzymes (DNMT3B, PFKFB4, SOD3, NT5DC2, PKM, GSTP1, SOAT2, FKBP10, and PYCR1) with positive individual coefficients (Figure 2D). Notably, the transcriptional expression of *DNMT3B*, followed by *PFKFB4* and *SOD3*, was identified as the most strongly correlated with positive metastatic status in hepatoblastoma (Figure 2D). The combined expression of these nine enzymes still allowed for accurate prediction of positive metastasis status, with an area under the curve of 0.86, sensitivity of 78.6%, and specificity of 87.2% (Figure 2E).

### 3.3. Tumor Co-Expression of DNMT3B and PFKFB4 as a Predictor of Metastasis and CHIC2 Risk Stratification in Hepatoblastoma

**Figure 3:**
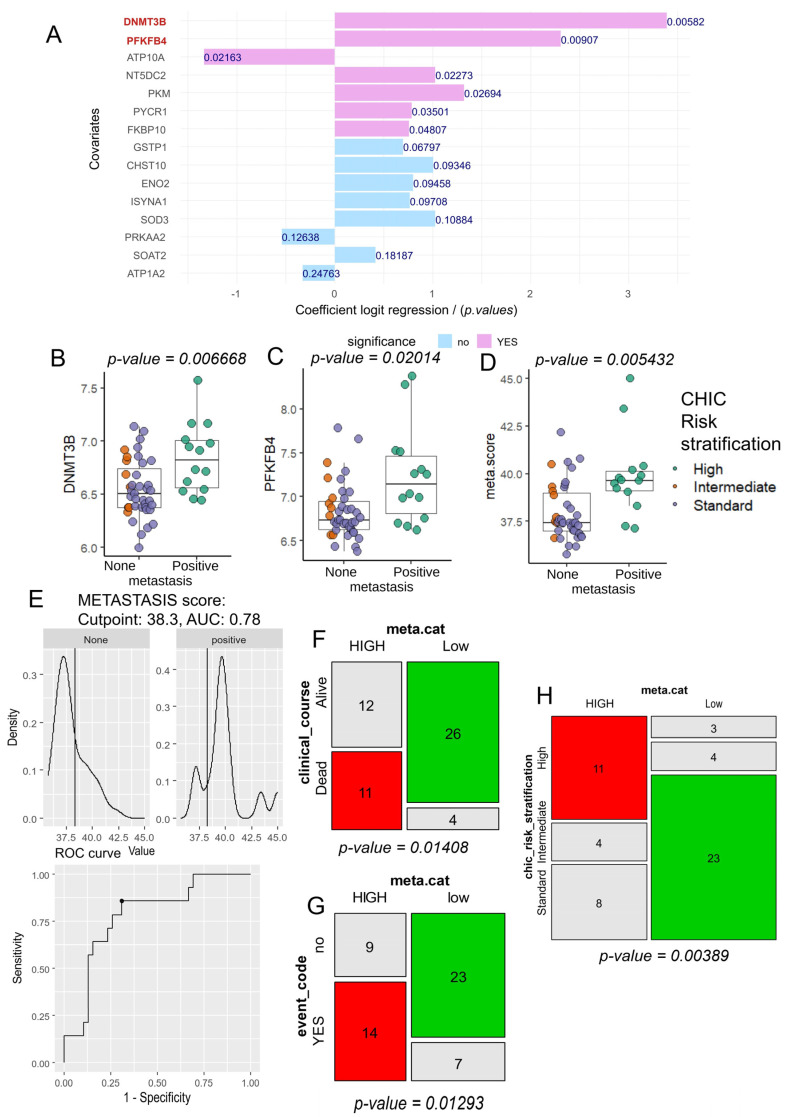
The combined expression of *DNMT3B* and *PFKFB4* as a predictor of metastasis and CHIC risk stratification in hepatoblastoma tumor evaluation. (**A**) Univariate binomial analyses to identify the best metabolic markers in hepatoblastoma tumors based on metastasis status; (**B**) a boxplot of *DNMT3B* expression, stratified by metastatic status and colored according to CHIC risk stratification, with *p*-values determined by a two-tailed *t*-test; (**C**) a boxplot of *PFKFB4* expression, stratified by metastatic status and colored according to CHIC risk stratification, with *p*-values determined by a two-sided *t*-test; (**D**) a boxplot of the metabolic/metastatic score (meta.score), which combines *DNMT3B* and *PFKFB4* expression, stratified by metastasis status and colored according to CHIC risk stratification, with *p*-values determined by a two-sided *t*-test; (**E**) determination of the optimal cutpoint for meta.score to predict metastasis status; (**F**) a mosaic plot showing the relationship between meta.score categories and clinical course status (*p*-value of chi-square test); (**G**) a mosaic plot showing the relationship between meta.score categories and clinical event (*p*-value of chi-square test); (**H**) a mosaic plot showing the relationship between meta.score categories and CHIC risk stratification (*p*-value of chi-square test).

A univariate logistic regression analysis was conducted for each enzyme in the HB metabolic-41 expression program, assessing the binary outcome of metastasis in hepatoblastoma (Figure 3A). These analyses confirmed that the transcriptional expression of DNMT3B and PFKFB4 in HB tumors is strongly associated with the prediction of metastatic status (Figure 3A). During univariate analyses against metastasis outcome, *DNMT3B* expression harbored an odds ratio of 29.64, and *PFKFB4* expression, an odds ratio of 10.07 (Table 2).

A significant difference in *DNMT3B* expression was indeed confirmed between hepatoblastoma (HB) tumors with negative and positive metastasis status by computational analysis (two-sided *t*-test *p*-value = 0.007; Figure 3B). The same was true for *PFKFB4* expression (two-sided *t*-test *p*-value = 0.020; Figure 3C). Based on the expression levels of these two markers, *DNMT3B* and *PFKFB4*, a metabolic score (meta.score) was calculated, and this score was found to be significantly different between HB tumors with positive and negative metastasis status (two-sided *t*-test, *p*-value = 0.0054; Figure 3D). ROC analysis was performed to determine the optimal threshold for the meta.score parameter in relation to metastatic status: a threshold of 38.3 yielded an area under the curve of 0.78 for predicting metastasis (Figure 3E). Using this meta.score threshold, the cohort was stratified into two groups: low (30 patients) and high (23 patients) (Table 1). Patients in these two groups did not show significant differences in terms of sex (*p*-value = 1; Table 1) or age at diagnosis (*p*-value = 0.27; Table 1). However, based on the meta.score stratification, significant differences were found in clinical course (*p*-value = 0.01408; Table 1), with a higher proportion of patients being alive in the low meta.score group (Figure 3F). Additionally, a significant difference was observed in clinical events during follow-up (*p*-value = 0.013; Table 1), with an increased proportion of positive clinical events in the high meta.score group (Figure 3G), as well as a significant difference in CHIC risk stratification.

### 3.4. Improved Metastasis Risk Assessment with the Combined DNMT3B and PFKFB4 Metabolic Expression Score

**Figure 4:**
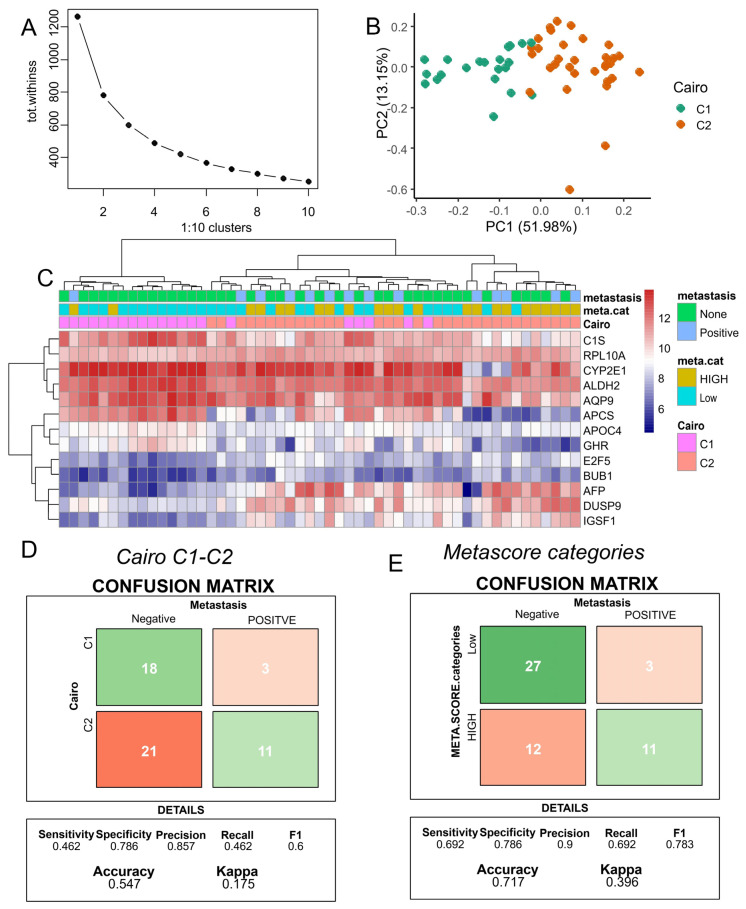
A comparative analysis of meta.score and C1–C2 classifier in the prediction of metastasis using dataset GSE131329. (**A**) The efficiency yield of cluster numbers during k-means clustering based on the C1–C2 expression signature; (**B**) principal component analysis with stratification of the C1/C2 group based on the Cairo signature; (**C**) unsupervised clustering of the C1/C2 signature, with stratification by Cairo prediction, metastasis status, and meta.score (metabolism); (**D**) a confusion matrix testing the accuracy of the Cairo C1–C2 classifier in predicting metastasis status; and (**E**) a confusion matrix testing the accuracy of meta.score in predicting metastasis status.

The 16-gene classifier for hepatoblastoma, as described by C1/C2 [23], distinguishes between two types of tumors with distinct expression patterns of hepatic stem/progenitor markers during their immature stages, related to the activation state of beta-catenin. We applied this reference classifier to the GSE131329 transcriptome dataset [9]. Given the Affymetrix Human Gene 1.0 ST Array technology used in this cohort, we were able to analyze 13 of the 16 markers. Based on the expression of these markers, a k-means classifier stratified the samples into two groups (Figure 4A), which were well-separated according to Cairo groups using principal component analysis (Figure 4B). Unsupervised clustering using Euclidean distances aggregated the majority of Cairo-C1 samples into the left cluster and the majority of Cairo-C2 samples into the right cluster (Figure 4C). A confusion matrix evaluating the prediction of metastasis status based on Cairo C1–C2 classification showed an accuracy of 0.55, with 46% sensitivity and 79% specificity (Figure 4D). In contrast, the meta.score allowed for an evaluation of metastasis prediction accuracy of 0.72, with 69% sensitivity and 79% specificity (Figure 4E). Notably, the meta.score outperformed the C1/C2 classifier in the prediction of metastasis status in hepatoblastoma in terms of accuracy and sensitivity, while the specificity of both parameters was found to be equivalent.

### 3.5. DNMT3B and PFKFB4 Metabolic Expression Score as an Independent Predictor of Metastasis in Hepatoblastoma

**Figure 5:**
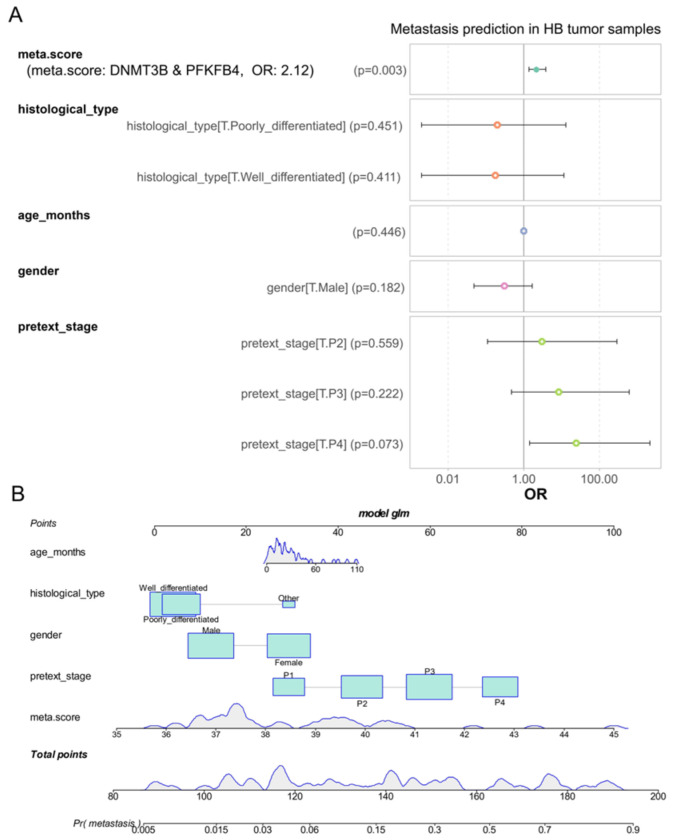
Meta.score as a novel, independent predictor of metastasis in hepatoblastoma tumors. (**A**) A forest plot of the regression binomial clinical-biological model with metastasis status as the outcome, incorporating distinct parameters: age at diagnosis (age_months), meta.score (combined expression of DNMT3B and PFKFB4), patient gender (with female as the reference category), histological type of tumor (with ‘other’ as the reference category), and PRETEXT stage (with stage 1 (P1) as the reference category, and subsequent stages denoted as P2: stage 2, P3: stage 3, and P4: stage 4). (**B**) A nomogram of the regression metastasis model, displaying odds ratios (OR).

We used a univariate analysis to demonstrate that the meta.score, derived from the combined expression of *DNMT3B* and *PFKFB4*, can predict metastasis status in hepatoblastoma tumors. This finding led us to construct a logistic regression model using the generalized linear model R function to address the binomial outcome of metastasis. The model incorporated meta.score expression, along with epidemiological parameters such as age at diagnosis and sex, as well as tissue differentiation and PRETEXT stages (Figure 5A). Notably, the meta.score continued to be a significant adverse factor in this regression model (*p*-value = 0.003, Figure 5A), with an odds ratio of 2.12 for predicting a positive metastatic status in hepatoblastoma. Furthermore, the nomogram for this model (Figure 5B) showed that meta.score values were well-represented within the total points, especially between 0 and 60. These findings suggest that, even after accounting for epidemiological, histological, and clinical parameters, the meta.score remains a significant and independent computational predictor of metastasis in hepatoblastoma.

## 4. Discussion

Our study, which utilized the Mammalian Metabolic Enzyme Database [17], led to the development of a metabolic score based on the combined expression of DNMT3B and PFKFB4 in hepatoblastoma. This score enabled the prediction of metastatic status and high-risk stratification in children with hepatoblastoma according to the Children’s Hepatic tumors International Collaboration (CHIC) risk stratification system. Notably, a significant proportion of high-risk CHIC hepatoblastoma patients exhibited distant metastasis and elevated expression of histone cluster genes and small nucleolar RNA, suggesting a correlation between distant metastasis and epigenetic regulation in hepatoblastoma [9].

Among the various epigenetic mechanisms, DNA methylation is a crucial process that involves the addition of a methyl group to cytosine (5mC) by DNA methyltransferase enzymes (DNMTs) [24]. In the context of hepatoblastoma, the enrichment of 5-hydroxymethylcytosine has been associated with disrupted expression of UHRF1, TET1, and TET2 [25]. DNMT3B, in conjunction with DNMT3A, plays a key role in de novo methylation, in contrast to DNMT1 [26]. In a study using the HCT116 colorectal cancer cell line, the disruption of DNMT1 and DNMT3B, as well as pharmacological inhibition with 5-Aza-2s-deoxycytidine (5-Aza-dC, decitabine), resulted in the demethylation of MEG3-DMR and the expression of 14q32 miRNAs, leading to the suppression of adhesion, invasion, and migration (AIM) properties in metastatic tumor cells [27]. Furthermore, in human adult hepatocellular carcinoma, microRNA-26a has been shown to inhibit cancer cell proliferation and metastasis by regulating the DNMT3B-MEG3 axis [28].

Phosphofructokinase 2 (PFK2) is a bifunctional enzyme with both kinase and phosphatase activities, encoded by four PFK2 isozymes in humans: PFKFB1, PFKFB2, PFKFB3, and PFKFB4 [29]. The phosphofructo-2-kinase/fructose-2,6-biphosphatase 4 (PFKFB4) isozyme has been implicated in tumor development through its regulation of glycolytic and pentose phosphate pathway flux, as well as ATP synthesis in hypoxic cells [30,31]. PFKFB4 has been induced by hypoxia in multiple cancer cell lines and overexpressed in matched human lung, breast, and colon tumor tissues relative to normal tissues from the same patients [32,33]. In adult hepatocellular carcinoma, PFKFB4 has been identified as a metabolic driver of disease progression and chemoresistance through its mitigation of reactive oxygen species (ROS) [34]. PFKFB4 has also been implicated in the metastatic process during cancer progression. In breast cancer, hypoxia-induced PFKFB4 in the tumor microenvironment has been shown to shape metabolic and cellular plasticity, increasing metastatic competence [35]. Furthermore, the Warburg pathway enzyme PFKFB4 has been found to couple sugar metabolism with transcriptional activation by stimulating SRC-3 and promoting aggressive metastatic tumors [36]. In melanoma, PFKFB4 has been shown to activate the RAS/AKT pathway, impacting cell migration [37]. PFKFB4 expression has also been found to be increased by carbonic anhydrase IX, promoting motility in human cervical cancer cells [38].

While our study identified *DNMT3B* and *PFKFB4* as individual predictive biomarkers of metastasis in hepatoblastoma, it is also possible that these two proteins interact or influence each other’s functions. Epigenetic regulation and metabolic pathways are known to be interconnected, and there is evidence to suggest that DNMT3B and PFKFB4 may interact in a manner that contributes to the development and progression of cancer. DNA methylation, which is mediated by DNMT3B, has been shown to regulate the expression of genes involved in glycolysis, including those that code for enzymes such as phosphofructokinase-1 (PFK-1) [39]. PFKFB4, which is a regulator of glycolysis, has been found to be overexpressed in various types of cancer, including hepatocellular carcinoma [34]. The promoter region of the PFKFB4 gene contains several CpG islands, which are potential targets for DNMT3B-mediated methylation [40]. It is possible that DNMT3B regulates the expression of PFKFB4 by methylating its promoter region, thereby modulating glycolytic activity in cancer cells. In addition to direct transcriptional regulation, DNMT3B and PFKFB4 may also interact indirectly through other signaling pathways. For example, DNMT3B has been shown to be regulated by the expression of the phosphatidylinositol 3-kinase (PI3K)/protein kinase B (AKT) pathway, which is also involved in the regulation of glycolysis [41]. PFKFB4 has been found to activate the AKT pathway, which in turn promotes cell survival and proliferation in cancer cells [37]. Therefore, it is possible that DNMT3B and PFKFB4 interact through the PI3K/AKT pathway to regulate glycolytic activity and promote cancer progression. Furthermore, recent studies have shown that epigenetic regulators such as DNMT3B can interact with metabolic enzymes, including those involved in glycolysis, to regulate cellular metabolism [39]. These interactions can have significant effects on cell growth, proliferation, and survival, and may contribute to the development of cancer.

In conclusion, our study has identified *DNMT3B* and *PFKFB4* as potential individual biomarkers of metastasis in hepatoblastoma. Additionally, there is some evidence to suggest that these two proteins may interact, directly or indirectly, to regulate glycolytic activity and contribute to cancer progression. While these preliminary results from scRNAseq data analysis are intriguing, further experimental validation is needed to fully understand the relationship between DNMT3B and PFKFB4. Nonetheless, these findings provide a promising starting point for investigating potential therapeutic strategies, including the possibility of targeting both proteins simultaneously, although more research is required to determine the efficacy of this approach in treating hepatoblastoma.

## 5. Conclusions

Our study identifies *DNMT3B* and *PFKFB4* as genes associated with metastatic characteristics in hepatoblastoma, suggesting them as potential biomarkers that require further investigation. These findings highlight the relevance of transcriptional profiling to uncover genes linked to tumor aggressiveness, although additional studies are necessary to confirm the predictive value of *DNMT3B* and *PFKFB4* in clinical settings. Future research should explore these targets in preclinical and clinical models to assess their utility in therapeutic strategies, with the aim of developing approaches that could improve outcomes in pediatric hepatoblastoma.

## Figures and Tables

**Table 1:** Clinical stratification (low and HIGH metabolic categories) of hepatoblastoma patients according to their metabolic score on metastasis (GSE131329).

Variable	Level	Low (*n* = 30)	HIGH (*n* = 23)	Total (*n* = 53)	*p*-Value
age_months	Mean (sd)	24 (22.8)	31.4 (25.7)	27.2 (24.1)	0.26766
CHIC_risk_stratification	Standard	23 (76.7)	8 (34.8)	31 (58.5)	
	High	3 (10.0)	11 (47.8)	14 (26.4)	
	Intermediate	4 (13.3)	4 (17.4)	8 (15.1)	0.00389
clinical_course	Alive	26 (86.7)	12 (52.2)	38 (71.7)	
	Dead	4 (13.3)	11 (47.8)	15 (28.3)	0.01408
Clinical event during follow up	No	23 (76.7)	9 (39.1)	32 (60.4)	
	YES	7 (23.3)	14 (60.9)	21 (39.6)	0.01293
histological_type	Well diff.	17 (56.7)	13 (56.5)	30 (56.6)	
	Other	1 (3.3)	1 (4.3)	2 (3.8)	
	Poorly diff.	12 (40.0)	9 (39.1)	21 (39.6)	0.98116
Sex	Female	14 (46.7)	11 (47.8)	25 (47.2)	
	Male	16 (53.3)	12 (52.2)	28 (52.8)	1.00000
PRETEXT stage	P3	10 (33.3)	8 (34.8)	18 (34.0)	
	P2	10 (33.3)	5 (21.7)	15 (28.3)	
	P4	4 (13.3)	7 (30.4)	11 (20.8)	
	P1	6 (20.0)	3 (13.0)	9 (17.0)	0.41827

**Table 2:** Univariate binomial analyses of the expression of the best metabolic markers to predict metastasis status in hepatoblastoma tumors.

Predictors	Beta Coefficients	Odds Ratios	*p*-Values
DNMT3B	3.389	29.638	5.82 × 10^−3^
PFKFB4	2.310	10.071	9.07× 10^−3^
NT5DC2	1.030	2.801	2.27× 10^−2^
PKM	1.321	3.745	2.69 × 10^−2^
PYCR1	0.792	2.208	3.50 × 10^−2^
FKBP10	0.764	2.146	4.81 × 10^−2^
GSTP1	0.702	2.017	6.80 × 10^−2^
CHST10	1.009	2.742	9.35 × 10^−2^
ENO2	0.802	2.230	9.46 × 10^−2^
ISYNA1	0.772	2.163	9.71 × 10^−2^

## Data Availability

The R-scripts used during this study are available at the following address: https://github.com/cdesterke/scripts_hb_metastasis (accessed on 9 September 2024).

## Supplementary Materials

The following supporting information can be downloaded at: https://www.mdpi.com/article/10.3390/biom14111394/s1, Table S1: metabolic-41 signature in hepatoblastoma tumors.
