# Correction: Desterke et al. Single-Cell RNA-Seq Analysis Links DNMT3B and PFKFB4 Transcriptional Profiles with Metastatic Traits in Hepatoblastoma. *Biomolecules* 2024, *14*, 1394

**DOI:** 10.3390/biom15060769

**Published:** 2025-05-27

**Authors:** Christophe Desterke, Raquel Francés, Claudia Monge, Agnès Marchio, Pascal Pineau, Jorge Mata-Garrido

**Affiliations:** 1 Faculté de Médecine du Kremlin Bicêtre, Université Paris-Sud, Université Paris-Saclay, 94270 Le Kremlin-Bicêtre, France; christophe.desterke@inserm.fr; 2 Cell and Tissue Biology Group, Anatomy and Cell Biology Department, University of Cantabria-IDIVAL, 39011 Santander, Spain; 3 International Joint Laboratory of Molecular Anthropological Oncology (LOAM), IRD, INEN, Lima 15038, Peru

## Affiliations

In the published publication [1], there was an error regarding the affiliations for Raquel Francés, Claudia Monge, Agnès Marchio, Pascal Pineau and Jorge Mata-Garrido. As a replacement for affiliations 2 and 3, as well as correspondence email, the updated affiliations should include the following:

^2^  Cell and Tissue Biology Group, Anatomy and Cell Biology Department, University of Cantabria-IDIVAL, 39011 Santander, Spain
^3^  International Joint Laboratory of Molecular Anthropological Oncology (LOAM), IRD, INEN, Lima 15038, Peru

In addition, the corresponding author, Jorge Mata-Garrido, moves from affiliation 3 to the new affiliation 2. * Correspondence: jmatag@unican.es.

All emails, except for christophe.desterke@inserm.fr and the correspondence email, must, in turn, be removed from the article.

## Funding

In the original publication [1], the funder “MEAE AMBASS FRANCE AU PEROU FSPI—S-AC23007, Filière Santé Maladie Rare du Foie de l’Adulte et de l’Enfant” must be modified to “This research received no external funding”, due to the fact that, since it is a study involving computation, the use of funds was not necessary.

The authors state that the scientific conclusions are unaffected. This correction was approved by the Academic Editor. The original publication has also been updated.
